# Taurine Prevents Impairments in Skin Barrier Function and Dermal Collagen Synthesis Triggered by Sleep Deprivation-Induced Estrogen Circadian Rhythm Disruption

**DOI:** 10.3390/cells14100727

**Published:** 2025-05-16

**Authors:** Qi Shao, Zhaoyang Wang, Yifang Li, Xun Tang, Ziyi Li, Huan Xia, Qihong Wu, Ruxue Chang, Chunna Wu, Tao Meng, Yufei Fan, Yadong Huang, Yan Yang

**Affiliations:** 1 State Key Laboratory of Bioactive Molecules and Druggability Assessment, Guangdong Basic Research Center of Excellence for Natural Bioactive Molecules and Discovery of Innovative Drugs, College of Life Science and Technology, Jinan University, Guangzhou 510632, China; astra@stu2022.jnu.edu.cn (Q.S.); wzy1003@stu2021.jnu.edu.cn (Z.W.); lyf0302@stu2023.jnu.edu.cn (Y.L.); xun3629487@stu2023.jnu.edu.cn (X.T.); lzy2023@stu2023.jnu.edu.cn (Z.L.); xiahuan@stu2019.jnu.edu.cn (H.X.); wuqihong2023@stu.jnu.edu.cn (Q.W.); chang2023@stu.jnu.edu.cn (R.C.); wuchunna@stu2023.jnu.edu.cn (C.W.); taoyhust@stu2022.jnu.edu.cn (T.M.); fanyf75@stu2023.jnu.edu.cn (Y.F.); 2 National Engineering Research Center of Genetic Medicine, Guangzhou 510632, China; 3 Guangdong Province Key Laboratory of Bioengineering Medicine, Guangzhou 510632, China; 4 TYRAN Cosmetics Innovation Research Institute, Jinan University, Guangzhou 511447, China

**Keywords:** taurine, sleep deprivation, estradiol circadian rhythm, epidermal barrier, collagen synthesis

## Abstract

Sleep deprivation is a prevalent issue that disrupts the circadian rhythm of estrogen, particularly estradiol, thereby significantly affecting women’s skin health and appearance. These disruptions can impair skin barrier functionality and decrease dermal collagen synthesis. In this study, our results demonstrate that topical taurine supplementation promotes the expression of tight junction (TJ)-related proteins and enhances collagen production, effectively restoring skin homeostasis in sleep-deprived female mice. Mechanistically, taurine upregulates the expression of *TMEM38B*, a gene encoding the TRIC-B trimeric cation channel, resulting in increased intracellular calcium ion levels. This, in turn, promotes the upregulation of TJ-related proteins, such as ZO-1, occludin, and claudin-11 in epidermal cells, while also enhancing the expression of type III collagen in fibroblasts, thus restoring skin homeostasis. These findings suggest that taurine may serve as an alternative to estradiol, effectively improving skin homeostasis disrupted by sleep deprivation while mitigating the potential risks associated with exogenous estrogen supplementation. Collectively, these results provide preliminary insights into the protective mechanisms of taurine against sleep deprivation-induced skin impairments and establish a foundation for its potential application in treating skin conditions related to estrogen imbalances, such as skin aging in menopausal women.

## 1. Introduction

Skin serves as a crucial barrier for the human body, providing isolation from the external environment while participating in moisture regulation, immune defense, and protection against pathogens [1]. The skin is structurally composed of two principal layers, the epidermis and dermis, with the epidermis further stratified into the stratum corneum (SC), stratum granulosum (SG), stratum spinosum (SS), and stratum basale (SB) from the exterior inward. The skin barrier is established through keratinocyte differentiation in the basal layer, culminating in the formation of the stratum corneum, the outermost protective layer of dead cells, accompanied by the production of specific molecular markers indicative of differentiation status [2,3]. A series of differentiation markers, including keratins KRT5 and KRT14 in the basal layer [4], KRT1 and KRT10 in the spinous layer [5], and envelope proteins loricrin (LOR) and profilaggrin (proFLG) in the granular layer [2], are critical for the barrier function of the epidermis. Tight junctions within the SG, composed of proteins such as claudins (CLDN), occludin (OCC), and the zonula occludens (ZO) family, regulate paracellular permeability and play a critical role in maintaining skin homeostasis [6]. Collagen, the most abundant protein in the dermis, is predominantly composed of type I and type III collagens, which are densely arranged and combined with elastin to create a resilient matrix that ensures mechanical strength and elasticity for the skin [7].

In contemporary society, the sleep deprivation-induced impairment of the skin barrier function has become a pressing concern affecting daily life. Epidemiological studies have indicated that sleep deprivation (SD) is highly prevalent, with more than one-third of adults in the Americas, Europe, and Asia reporting fewer than the recommended 7 h of sleep per night [8]. Chronic sleep insufficiency can disrupt circadian rhythms, particularly in women, who experience higher rates of sleep disturbances, such as insomnia and other sleep disorders [9]. Notably, circadian rhythm oscillations have been observed in both epidermal keratinocytes and dermal fibroblasts [10,11]. The differentiation of keratinocytes depends on the expression of clock genes, and disruptions in these oscillations can impair the division of epidermal stem cells, compromising epidermal homeostasis [12]. Additionally, circadian rhythms significantly influence collagen synthesis and the migration and adhesion of fibroblasts [13,14]. Moreover, sleep deprivation-induced circadian rhythm disruption can disturb systemic hormonal balance, particularly affecting estradiol homeostasis, which is closely linked to skin barrier integrity [15,16].

Estradiol secretion, regulated by the hypothalamus-pituitary-ovarian (HPO) axis, exhibits significant circadian rhythmicity [17]. Circadian clock imbalances disrupt the rhythmic expression of clock genes, significantly affecting estradiol secretion patterns [15]. Moreover, estradiol plays a critical role in maintaining skin health [16]. Estrogen deficiency accelerates skin changes typically attributed to extrinsic aging, such as dryness, atrophy, fine lines, and poor wound healing [18,19]. In the skin, type I (80%) and type III (15%) collagen levels are the most prevalent and are closely related to estrogen levels [20]. Systemic hormone replacement therapy and topical estrogens have been shown to increase collagen synthesis [21,22,23]. Despite these positive effects, the use of exogenous estrogen may increase the risk of breast, ovarian, and endometrial cancers [24,25,26]. Therefore, exploring alternatives to estrogen that can improve sleep deprivation-induced impaired skin homeostasis is of significant importance.

Taurine, a sulfur-containing amino acid derivative present in most mammalian tissues, plays roles in maintaining cell homeostasis through osmoregulation, antioxidant, anti-inflammatory, protein-stabilizing, and calcium-regulating actions [27]. Taurine is primarily distributed in the epidermis and modulates skin moisture content [28]. Its distribution in the skin, particularly in proximity to tight junctions, suggests a potential role in regulating tight junction function [29]. Notably, taurine not only attenuated UVB-induced wrinkle formation but also enhanced the synthesis of key epidermal components, such as ceramide and filaggrin, along with hyaluronic acid in the dermal extracellular matrix, consequently decreasing transepidermal water loss (TEWL) [30,31]. Moreover, the beneficial effects of taurine on collagen content and properties may provide a potential strategy for delaying skin aging [32,33]. However, the role of taurine in mitigating sleep deprivation-induced impaired skin homeostasis and its molecular mechanisms remains to be elucidated.

In this study, we demonstrated that taurine could substitute for estradiol in alleviating impairments in skin barrier function and dermal collagen synthesis caused by sleep deprivation-induced disruptions in estradiol circadian rhythms. This effect is mediated through the regulation of trimeric cation channels (TRICs), particularly the TRIC-B subtype encoded by *TMEM38B*. Our findings not only provided preliminary insights into the protective mechanisms of taurine against skin homeostasis impairments induced by sleep deprivation but also established an experimental foundation for its potential application in treating skin diseases associated with estradiol imbalances, such as skin aging in women during menopause.

## 2. Materials and Methods

### 2.1. Cell Culture and Drug Delivery Treatments

The human keratinocyte cell line (HaCaT) and mouse fibroblast cell lines (NIH 3T3) were obtained from Pronocell Biotech Co., Ltd. (Wuhan, China). Cells were grown in Dulbecco’s Modified Eagle Medium (DMEM) (C11995500BT, Gibco, Grand Island, NY, USA) supplemented with 10% fetal bovine serum (FBS) (FSD500, ExCell Bio, Shanghai, China). The cells were cultivated at 37 °C in a humidified environment with 5% CO_2_ in a cell culture incubator. Cell passages were digested using 0.25% trypsin (25200072, Gibco, Grand Island, NY, USA). Estradiol (HY-B0141, MedChemExpress, Monmouth Junction, NJ, USA) was administered at concentrations of 0.1, 1, and 10 nM for 24 h, while taurine (HY-B0351, MedChemExpress, Monmouth Junction, NJ, USA) was administered at concentrations of 0.1, 1, and 10 μM for 24 h. Fulvestrant (HY-13636, MedChemExpress, Monmouth Junction, NJ, USA) at 1 μM was added 4 h prior to the administration of other drugs, which were applied concomitantly in the presence of fulvestrant. Additionally, the cells were starved for 12 h in DMEM without FBS before administering the drug treatments.

### 2.2. Quantitative Real-Time PCR (qRT-PCR)

Tissues and cells were treated with TRIzol reagent (15596026, Invitrogen, Carlsbad, CA, USA) to extract total RNA. A reverse transcription kit (RR036A, Takara, Tokyo, Japan) was then used to transform the RNA into cDNA. The ChamQ SYBR qPCR Master Mix (Q311-02, Vazyme, Nanjing, China) was used to conduct quantitative real-time PCR (qRT-PCR) on a CFX96 Real-Time PCR Detection System (Bio-Rad, Hercules, CA, USA). The reference gene, either *GAPDH* or *β-actin*, was used to standardize the levels of gene expression. To analyze the data, the 2^−ΔΔCt^ method was used. The primers for qPCR were designed based on coding sequences obtained from NCBI, using Primer-BLAST. Table 1 contains the list of primers used in qRT-PCR.

### 2.3. Western Blot Assay

RIPA lysis buffer (FD008, Fidelity Bio, Hangzhou, China) combined with 0.1 mM PMSF (FD0100, Fidelity Bio, Hangzhou, China) was used to extract proteins from cells and skin tissues. Protein supernatants were obtained by centrifugation at 12,000 rpm, and protein content was quantified using the BCA Protein Assay Kit (23225, Thermo Fisher Scientific, Waltham, MA, USA). SDS-PAGE was used to separate the proteins, which were then moved to a PVDF membrane (10600021, Cytiva, Marlborough, MA, USA) and blocked for 1 h at room temperature using 5% bovine serum albumin (BSA) (9048-46-8, Aladdin, Ontario, CA, USA). After that, primary antibodies were placed on the membrane for an entire night at 4 °C, and secondary antibodies were incubated for 1 h at room temperature. Primary and secondary antibodies are listed in Table 2. Target proteins were visualized using ECL Western Blotting substrate (K12045D50, Advansta, San Jose, CA, USA). The intensity of the bands for each protein was quantified using Image J software (1.49v, National Institutes of Health, Bethesda, MD, USA).

### 2.4. Transepithelial Electrical Resistance (TEER) Measurements

HaCaT cells were seeded at a density of 1 × 10^5^ cells per well onto Transwell^®^ chambers with a 0.4 µm pore size polyester membrane (3470, Corning Incorporated, Corning, NY, USA) and treated in accordance with different experimental requirements. The integrity of the cell monolayer was assessed by measuring the Transepithelial Electrical Resistance (TEER) using the Millicell ERS system (Millipore, Bedford, MA, USA). The TEER value for each sample was calculated as follows: TEERsample (Ω·cm^2^) = (Rsample − Rblank) (Ω) × effective membrane area (cm^2^).

### 2.5. Knockdown and Overexpression

The pcDNA3.1 overexpression plasmid was constructed and transfected for 48 h using Lipofectamine 3000 (L3000015, Thermo Fisher, Waltham, MA, USA) as the transfection reagent according to the instruction manual. Lipofectamine RNAiMAX (13778100, Thermo Fisher, Waltham, MA, USA) was used to deliver siRNA for 60 h. Table 3 lists the primers used.

### 2.6. Calcium Ion Content Detection Assay

HaCaT and NIH 3T3 cells were inoculated into 96-well plates and treated with different concentrations of test compounds for 12 h. Calcium levels were measured using a calcium detection assay kit (36310, AAT Bioquest, Pleasanton, CA, USA) according to the manufacturer’s instructions. After treatment with Calbryte^TM^520AM (1:500 dilution) for 30 min at 37 °C, free Ca^2+^ was washed with HHBS. Changes in calcium ion fluorescence were immediately detected in real time using a full-featured fluorescence enzyme marker (Synergy H1). For stimulation of Ca^2+^ release, 200 μM adenosine triphosphate (ATP) was injected into cultures. Photographs were taken using a laser scanning confocal microscope (Zeiss, LSM 900, Oberkochen, Germany) at an excitation wavelength of 529 nm.

### 2.7. Immunofluorescence Assay

HaCaT and NIH 3T3 cells were inoculated into 20 mm diameter confocal culture dishes at a density of 1.5 × 10^5^ cells per dish. After attachment, cells were treated with estradiol, taurine, or calcium-free medium (Ca^2+^ free) for 24 h. Following a 10-min fixation with 4% paraformaldehyde (PFA), the cells were rinsed three times for 3 min each with PBS. At room temperature, they were blocked for 1 h using 5% BSA. Subsequently, primary antibodies against ZO-1 (Ab221547, Abcam, Cambridge, MA, USA), occludin (Ab216327, Abcam, Cambridge, MA, USA), and Collagen III (EPR17673, Abcam, Cambridge, MA, USA) at a dilution of 1:200 were incubated at 4 °C overnight. Following a PBS wash, cells were treated for 1 h at room temperature with Alexa Fluor^®^ 594 (Ab150080, Abcam, Cambridge, MA, USA) or Alexa Fluor^®^ 488 (Ab150077, Abcam, Cambridge, MA, USA) secondary antibodies (dilution 1:200). The nuclei were then stained with DAPI (AR1177, Boster Bio, Pleasanton, CA, USA). Lastly, the LSM900 confocal microscope (Zeiss, Oberkochen, Germany) was used to observe the cells.

Animal Skin Tissue: After the mice were euthanized, the dorsal skin tissues were embedded in OCT compound (62534, Sakura Finetek, Tokyo, Japan), after which the skin was cut into 8 μm sections using a cryostat. After 5 min of acetone fixation, the sections were washed with PBS and underwent a process of blocking, primary antibody, and secondary antibody incubation. The nuclei were stained with DAPI. The LSM900 (Zeiss, Oberkochen, Germany) was used to observe the fluorescence.

### 2.8. Animal Experiment

The 8-week-old female *Kunming* ***(****KM)* mice used in this experiment were purchased from the Animal Center of Guangdong Medical Laboratory (Guangdong, China) and were maintained on a 12-h light/dark cycle (24 ± 2 °C) with a standard rodent diet and drinking water available free of charge. The mice were acclimatized to this environment for one week. In the first part of the study, mice were divided into two groups by means of simple randomization for sampling skin and serum during the day and night (15:00 and 21:00, respectively, n = 7). In the second part, the mice were divided into three groups by simple randomization: control, sleep deprivation (SD), and SD with taurine administration (n = 7 per group). Sleep deprivation was induced using the modified multiplatform method (MMPM), a classical technique based on the principle that muscle weakness during the rapid eye movement stage of sleep causes mice to fall into water and wake up [34,35]. This method allows sleep deprivation of multiple animals simultaneously without affecting other physiological activities. The MMPM apparatus consisted of a large water tank (485 × 350 × 200 mm) containing 15 cylindrical platforms (3 cm in diameter and 5 cm in height), spaced 5 cm apart both vertically and horizontally. The platforms were elevated 1 cm above the water surface, and the tank was covered with an iron mesh to allow normal feeding. Mice were sleep-deprived from 17:00 to 11:00 the following day, with a 6-h recovery period, for a total duration of 35 days. In the SD + taurine group, beginning on the 8th day of sleep deprivation, the dorsal hair of the mice was locally removed, and 100 μM taurine (dissolved in water containing 1% carbomer) was topically applied to the depilated area. At the end of the experiment, blood samples were collected from the tail at 15:00, and orbital blood and skin tissues were collected at 21:00. Skin tissues were embedded in OCT for immunofluorescence analysis, fixed in 4% PFA for 24 h for subsequent hematoxylin-eosin, Masson’s trichrome, and Sirius red staining, or frozen at −80 °C for Western blot and qRT-PCR assays. Considering the impact of physiological rhythms on estrogen levels, the estrous cycle of animals was monitored, and sampling was performed outside the estrous phase of female mice to ensure accurate results. To maintain the integrity of the blind experiment, distinct individuals were assigned to each of the following roles: group assignment, experiment execution, results evaluation, and data analysis. All animal experiments were conducted in accordance with the National Institutes of Health Animal Care and Use Guidelines and approved by the Institutional Animal Care and Use Committee of Jinan University (Approval No. IACUC-20210630-04).

### 2.9. FITC Penetration Assessment of the Skin Barrier

FITC was dissolved in anhydrous DMSO to a concentration of 1 mg/mL, diluted to 10 μg/mL with PBS, and 400 μL of it was applied to the back of hairless mice using a sterile dressing. The whole process was protected from light. After 4 h, the mice were euthanized, and the skin tissue was embedded in OCT and sectioned at a thickness of 8 μm. The slices were fixed in acetone for 5 min, then washed with PBS and stained with DAPI for 10 min to display the cell nuclei. Finally, the slices were inspected with a fluorescence microscope (Nikon, Tokyo, Japan).

### 2.10. Hematoxylin-Eosin Staining

Tissue samples were embedded in paraffin and sectioned. Sections were dewaxed in xylene (15 min, twice), followed by a 1:1 mixture of xylene and anhydrous ethanol (2 min), 100% ethanol (5 min, twice), and 80% ethanol (5 min). Rehydration was completed by rinsing in distilled water for 5 min. Next, the sections were stained with hematoxylin for 5 min, rinsed in distilled water, differentiated in 1% hydrochloric acid alcohol for 30 s, and returned to blue in a weak alkaline solution. Eosin staining (0.5%) was performed for 3 min, followed by rinsing in distilled water. Then, dehydration was carried out in 80% ethanol (30 s), 95% ethanol (1 min, twice), and anhydrous ethanol (3 min, twice). Finally, the sections were immersed in xylene (3 min, twice) and sealed with neutral gum.

### 2.11. Masson Staining

Paraffin sections were dewaxed in xylene three times (5 min each) and rehydrated through anhydrous ethanol, 95% ethanol, and 75% ethanol (1 min each), followed by rinsing with tap and distilled water. Sections were stained with Weigert’s iron hematoxylin for 8 min, differentiated in 1% hydrochloric acid alcohol (15 s), blued with Masson blue solution (5 min), and rinsed with distilled water. The sections were stained with Masson Ponceau acid fuchsin (10 min) and washed with 2% glacial acetic acid (1 min). Differentiation was done with 1% phosphomolybdic acid (3 min), followed by rinsing in 2% glacial acetic acid, staining with aniline blue (1 min), and washing with 0.2% glacial acetic acid. Ultimately, the sections were dehydrated in ethanol, cleared in xylene, and mounted with neutral resin for microscopic examination.

### 2.12. Sirus Red Stain

Paraffin sections were dewaxed, followed by staining with iron hematoxylin for 10 min. Excess stain was removed by rinsing with distilled water. The sections were then stained with Sirius red for 20 min and gently rinsed with running water to remove excess stain. For dehydration and transparency, the sections were sequentially incubated in 75% ethanol, 95% ethanol, and anhydrous ethanol (1 min each), followed by clearing in xylene three times. The slides were covered with neutral resin and observed under a microscope.

### 2.13. Serum Estradiol Assay

To measure serum estradiol levels, blood samples were collected at different time points. Centrifugation at 3500× *g* for 15 min was used to separate the serum and plasma. As directed, an ELISA kit (Elabscience, Wuhan, China) was used to assess the serum estradiol levels.

### 2.14. Statistical Analysis

GraphPad Prism 8’s one-way ANOVA or *t*-test was used to evaluate mean differences, and data were displayed as mean ± standard deviation (SD) from a minimum of three independent trials. Differences were considered statistically significant at * *p* < 0.05; ** *p* < 0.01; *** *p* < 0.001; **** *p* < 0.0001.

## 3. Results

### 3.1. The Alterations of Skin Barrier and Collagen Production Under the Influence of Circadian Rhythm

**Figure 1:**
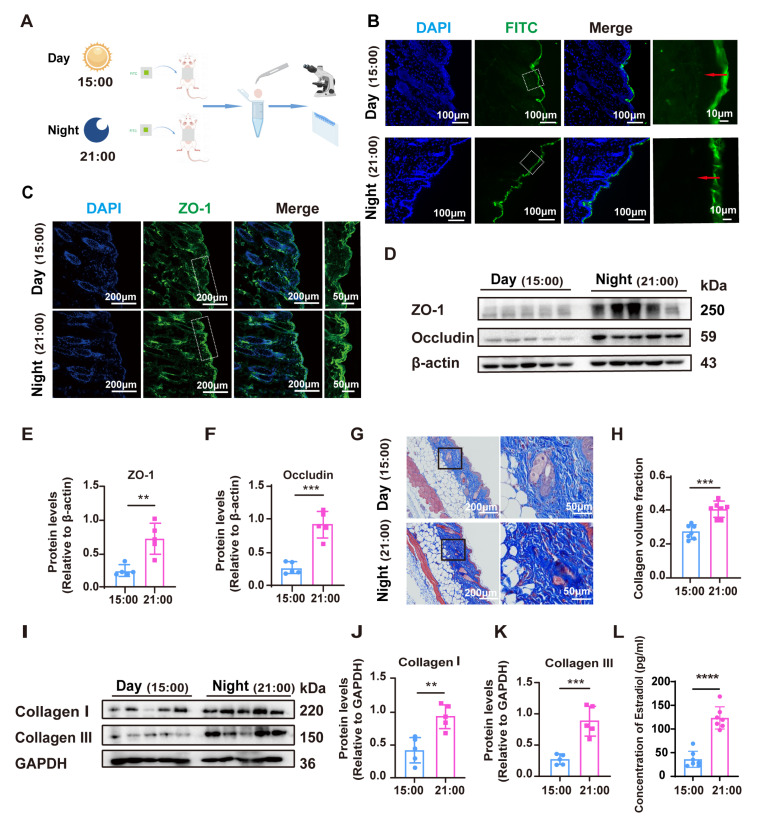
The alterations of skin barrier and collagen production under the influence of circadian rhythm. (**A**). Schematic diagram of the animal experiment. (**B**). FITC (green) permeability for assessing skin barrier integrity at 15:00 and 21:00. Nuclei were stained with DAPI (blue). The rightmost panel showed a magnified view of the highlighted area. Local permeability was highlighted by the red arrow. Scale bars, 100 μm and 10 μm. (**C**). Immunofluorescent staining for ZO-1 (green) at 15:00 and 21:00. Nuclei were stained with DAPI (blue). The rightmost panel showed a magnified view of the highlighted area. Scale bars, 200 μm and 50 μm. (**D**). Expression of ZO-1 and occludin was detected by Western blot at 15:00 and 21:00, (n = 5). (**E**,**F**). Quantitative analysis of relative expression levels of ZO-1 and occludin by ImageJ software (1.49v, National Institutes of Health, Bethesda, MD, USA). (**G**). Collagen volume fraction assessed by Masson staining at 15:00 and 21:00, (n = 7). The rightmost panel showed a magnified view of the highlighted area. Scale bars, 200 μm and 50 μm. (**H**). Quantitative analysis of collagen volume fraction in (**G**) by ImageJ software. The average of three fields of view was taken for each slice. (**I**). Expression of collagen I and collagen III was detected by Western blot at 15:00 and 21:00, (n = 5). (**J**,**K**). Quantitative analysis of relative expression levels of collagen I and collagen III by ImageJ software. (**L**). Detection of serum estradiol levels at 15:00 and 21:00, (n = 7). Data presented as means ± SD. ** *p* < 0.01, *** *p* < 0.001, **** *p* < 0.0001.

To investigate whether the barrier function of the mouse epidermis is influenced by circadian rhythms, we applied FITC to the depilated back skin of female mice at 15:00 (daytime) and 21:00 (nighttime) (Figure 1A). The integrity of the skin barrier was assessed by detecting the permeability of the FITC. The results showed that FITC was more effectively blocked on the skin surface at night (21:00), suggesting superior barrier integrity, whereas its penetration was more pronounced during the day (15:00) (Figure 1B). Further investigation using immunofluorescence for the tight junction protein ZO-1 at 15:00 and 21:00 revealed that ZO-1 was more localized in the epidermal layer at night, forming a more complete barrier, while its continuity and tightness were diminished during the daytime (Figure 1C). Additionally, Western blot analysis confirmed that the expression levels of tight junction (TJ)-associated proteins ZO-1 and occludin were higher at night compared to the daytime (Figure 1D–F). In addition, Masson staining results showed that the content of collagen in the skin was significantly higher at night than during the day, indicating that the abundance of collagen was also affected by the circadian rhythm (Figure 1G,H). In order to confirm this conclusion, we detected the expression levels of collagen I and collagen III in skin tissues by Western blot. The results showed that the expression levels of collagen I and collagen III were significantly lower in the day than in the night, indicating that the abundance of collagen was influenced by circadian rhythm (Figure 1I–K). We mentioned that estradiol is an important hormone that affects skin barrier function. Interestingly, we found that the serum levels of estradiol in mice were lower during the day and higher at night, indicating that estradiol also has a certain circadian rhythm (Figure 1L).

These findings collectively suggest that both tight junction integrity and collagen content exhibit circadian rhythm-dependent fluctuations, with optimal barrier function and collagen production occurring at night. Notably, these rhythmic changes correspond with fluctuations in estradiol levels, implying that estradiol may play a crucial role in modulating epidermal barrier function and collagen synthesis.

### 3.2. Estradiol Promoted the Expression of Tight Junction (TJ)-Related Proteins and the Production of Collagen

**Figure 2:**
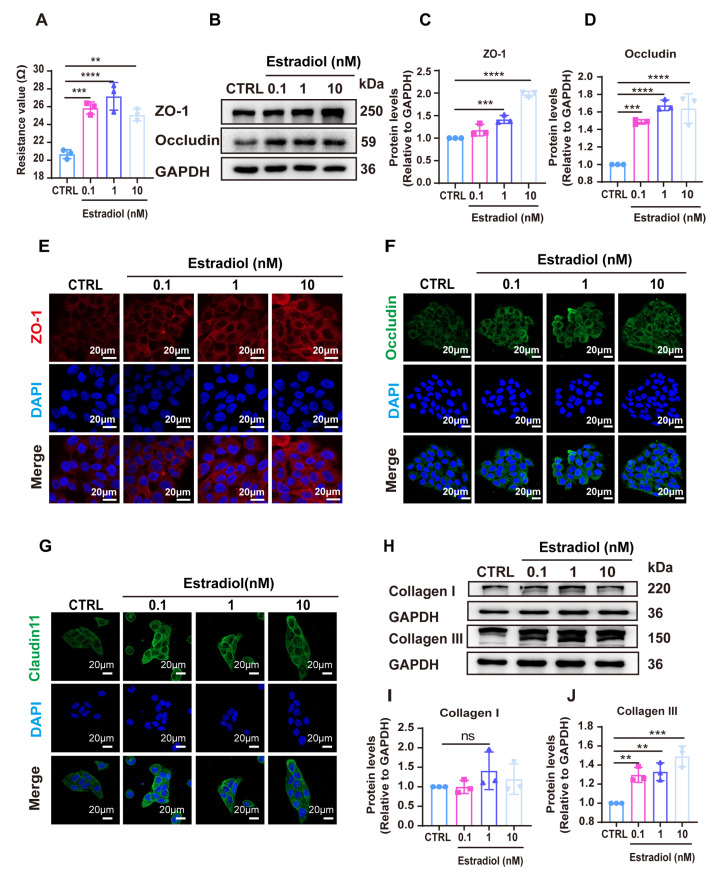
Estradiol promoted the expression of tight junction (TJ)-related proteins and the production of collagen. (**A**). TEER value of HaCaT monolayers after 24 h treatment with estradiol (n = 3). (**B**). Expression of ZO-1 and occludin was detected by Western blot after 24 h treatment with estradiol at different concentrations (n = 3). (**C**,**D**). Quantitative analysis of relative expression levels of ZO-1 and occludin by ImageJ software. (**E**–**G**). Immunofluorescence staining showed endogenous ZO-1 (red), occludin (green), and Claudin11 (green) after treatment with different concentrations of estradiol. Scale bars, 20 μm. (**H**). Expression of collagen I and collagen III was detected by Western blot after 24 h treatment with estradiol at different concentrations (n = 3). (**I**,**J**). Quantitative analysis of relative expression levels of collagen I and collagen III by ImageJ software. Data presented as means ± SD. ** *p* < 0.01, *** *p* < 0.001, **** *p* < 0.0001, ns = not significant.

To further investigate the role of estradiol in the formation of tight junctions and permeability in epidermal cells, we measured the trans-epithelial electrical resistance (TEER) across the epidermal cell monolayers. Given that keratinocytes are the predominant cell type in the epidermis, we treated HaCaT cell monolayers with estradiol at concentrations of 0.1 nM, 1 nM, and 10 nM. Our results indicated a significant increase in TEER values following estradiol treatment across all concentrations tested (Figure 2A). Western blot analyses also revealed that estradiol significantly upregulated the expression of ZO-1 and occludin, with the most pronounced effects observed at 1 nM and 10 nM (Figure 2B–D). Immunofluorescence results showed that after different concentrations of estradiol-treated HaCaT cells, the fluorescence intensity of barrier-related proteins ZO-1, occludin, and Claudin11 significantly increased (Figure 2E–G), indicating that estradiol can significantly promote the expression of ZO-1, occludin, and Claudin11 at the cellular level. It is suggested that estradiol has a potential role in promoting the formation of a skin barrier. In addition, in order to explore the effect of estradiol on collagen, we treated mouse fibroblasts with different concentrations of estradiol. The results showed that estradiol at 0.1 nM, 1 nM, and 10 nM significantly promoted the expression of type III collagen, while the expression of type I collagen had no significant changes (Figure 2H–J). These results suggest that estradiol is essential for the formation of collagen III.

Overall, these results suggested that the elevated estradiol levels at night enhance skin barrier integrity by promoting the expression of TJ-related proteins and the production of collagen III in mice. However, during the daytime, the estradiol levels decrease, leading to a reduced skin barrier function and decreased collagen synthesis.

### 3.3. Effects of Circadian Rhythm Disruption on Skin Barrier Function and Collagen Synthesis in Sleep-Deprived Mice

**Figure 3:**
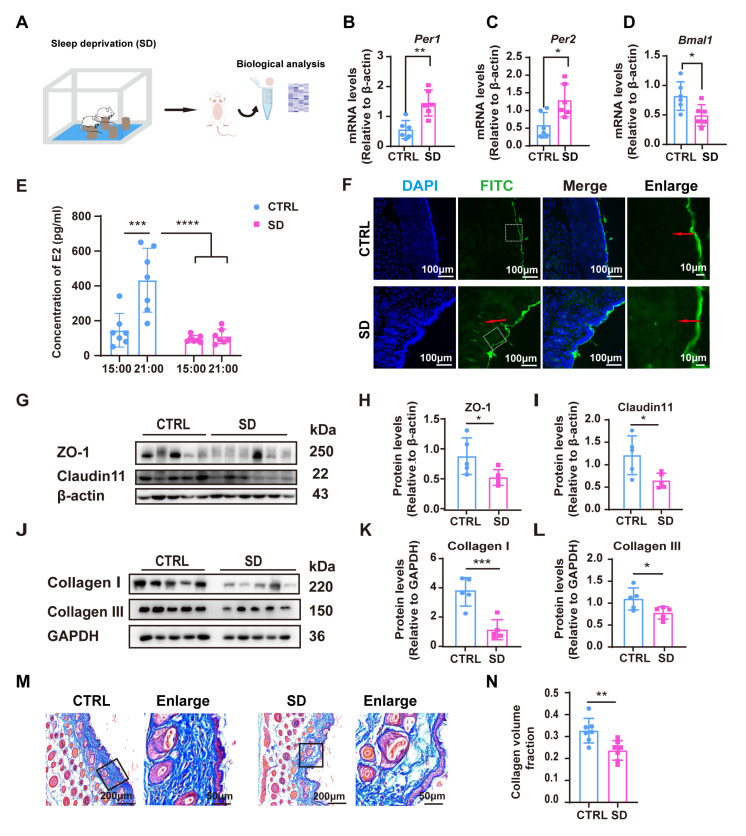
Effects of circadian rhythm disruption on skin barrier function and collagen synthesis in sleep-deprived mice. (**A**). Schematic diagram of the animal experiment. (**B**–**D**). Detection of mRNA levels of the rhythmic genes *Per1*, *Per2*, and *Bmal1* by qRT-PCR (n = 6). (**E**). Estradiol changes in the serum of mice at 15:00 and 21:00 were detected in different groups, respectively (n = 7). (**F**). FITC (green) permeability for assessing skin barrier integrity in control and SD. Nuclei were stained with DAPI (blue). The rightmost panel showed a magnified view of the highlighted area. Local permeability was highlighted by the red arrow. Scale bars, 100 μm and 10 μm. (**G**). Expression of ZO-1 and Claudin11 was detected by Western blot in control and SD (n ≥ 5). (**H**,**I**). Quantitative analysis of relative expression levels of ZO-1 and Claudin11 by ImageJ software. (**J**). Expression of collagen I and collagen III was detected by Western blot in control and SD (n = 5). (**K**,**L**). Quantitative analysis of relative expression levels of collagen I and collagen III by ImageJ software. (**M**). Collagen volume fraction assessed by Masson staining in control and SD, (n = 7). Scale bars, 200 μm. (**N**). Quantitative analysis of collagen volume fraction in (**M**) by ImageJ software. Data presented as means ± SD. * *p* < 0.05, ** *p* < 0.01, *** *p* < 0.001, **** *p* < 0.0001.

In order to investigate the effect of rhythm disturbance on skin barrier function, we disrupted the rhythm of mice by subjecting them to sleep deprivation (SD) (Figure 3A). To identify whether the circadian rhythm of mouse skin is disrupted, we detected the expression changes of core clock genes *Per1*, *Per2*, and *Bmal1* in skin tissue. The results showed that the mRNA levels of *Per1*, *Per2*, and *Bmal1* in the Ctrl group were significantly different from those in the SD group, suggesting that the circadian rhythm in the skin of mice is disorganized (Figure 3B–D), and it is feasible to construct a mouse model of circadian disturbance through sleep deprivation. Based on the SD model, we examined changes in estradiol. The results showed that estradiol levels were lower during the daytime (15:00) and elevated at nighttime (21:00). Conversely, in SD mice, estradiol levels showed no significant fluctuations between daytime and nighttime, indicating a loss of circadian rhythm (Figure 3E). Additionally, to investigate whether the barrier function of the mouse epidermis is influenced by SD, we applied FITC to the depilated back skin of mice. After 4 h, FITC was more effectively restricted on the skin surface in the Ctrl group, suggesting enhanced barrier integrity, while its penetration was more noticeable in regions with compromised barrier continuity in the SD group, with significant penetration observed in the dermis (Figure 3F). Further, Western blot results indicated that in comparison to the Ctrl group, SD significantly reduced the expression of TJ-related proteins ZO-1 and Claudin-11, as well as type I and type III collagen in the dermis (Figure 3G–L). Masson’s trichrome staining revealed distinct morphological alterations in dermal collagen architecture within the SD group. Specifically, collagen fibers exhibited a disorganized arrangement accompanied by the disruption of fibril bundle alignment, with notable fragmentation observed in localized regions. Quantitative analysis demonstrated collagen proportions were dramatically diminished compared to the control group (Figure 3M,N).

The findings indicated that sleep deprivation disrupted the circadian rhythm of estradiol, impaired the functionality of the tight junctions within the epidermal barrier, and diminished the synthesis of dermal collagen.

### 3.4. Taurine Restored the Expression of Tight Junction (TJ) Proteins and Collagen Production Reduced by Estradiol Deficiency

**Figure 4:**
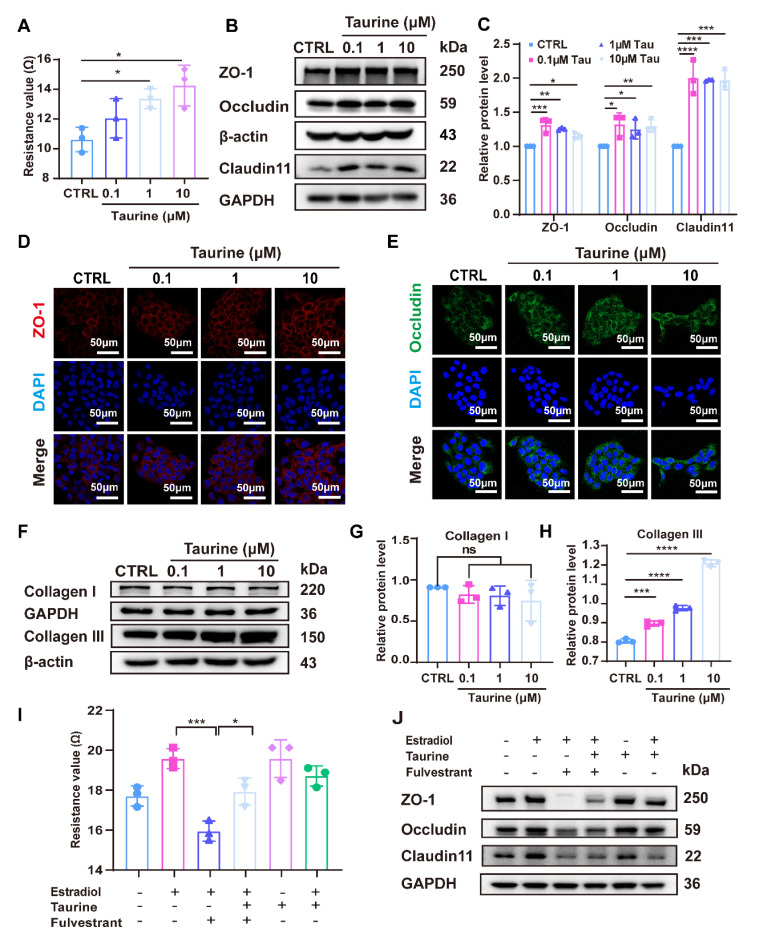
Taurine restored the expression of tight junction (TJ) proteins, and collagen production was reduced by estradiol deficiency. (**A**). TEER value of HaCaT monolayers after 24 h treatment with taurine. (**B**). Expression of ZO-1, occludin, and Claudin11 was detected by Western blot after 24 h treatment with taurine at different concentrations. (**C**). Quantitative analysis of relative expression levels of ZO-1, occludin, and Claudin11 by ImageJ software. (**D**,**E**). Immunofluorescence staining showed that endogenous ZO-1 (red) and occludin (green) after treatment with different concentrations of estradiol. Scale bars, 50 μm. (**F**). Expression of collagen I and collagen III was detected by Western blot after 24 h treatment with estradiol at different concentrations. (**G**,**H**). Quantitative analysis of relative expression levels of collagen I and collagen III by ImageJ software. (**I**). TEER value of HaCaT monolayers treated with different drugs for 12 h. (**J**). Expression of ZO-1, occludin, and Claudin11 was detected by Western blot after 12 h treatment with different drugs. The data were representative of at least three independent experiments and presented as means ± SD. * *p* < 0.05, ** *p* < 0.01, *** *p* < 0.001, **** *p* < 0.0001, ns = not significant.

Since the use of exogenous estrogens may increase the risk of cancer [24], it is necessary to explore a new class of drugs to treat skin barrier damage. Taurine, an amino acid present in the skin, has been introduced into research due to its reported protective effects on skin health [30]. To ensure the safety of taurine, we conducted a CCK8 assay to evaluate its cytotoxicity at various concentrations (0.1 μM, 1 μM, and 10 μM). The results demonstrated that taurine at these concentrations had no significant impact on cell viability, thereby confirming its safety profile (Figure S1). To investigate the role of taurine in skin barrier function, HaCaT cells were treated with various concentrations of taurine (0.1 μM, 1 μM, and 10 μM) for 24 h. The results indicated that taurine at both 1 μM and 10 μM significantly increased the trans-epithelial electrical resistance (TEER) values of HaCaT monolayers, suggesting enhanced barrier integrity (Figure 4A). Western blot analysis demonstrated a significant elevation in the expression of TJ proteins, including ZO-1, occludin, and Claudin-11, at 0.1 μM, 1 μM, and 10 μM taurine (Figure 4B,C). Subsequent immunofluorescence experiments further confirmed these findings by showing increased protein expression of ZO-1 and occludin in response to taurine treatment (Figure 4D,E). Additionally, the effects of taurine on collagen production were tested in fibroblasts. Taurine significantly promoted the expression of collagen III in a dose-dependent manner (Figure 4F–H).

To examine whether taurine can restore the skin barrier compromised by estradiol deficiency, we used fulvestrant, an estradiol receptor antagonist. Our findings demonstrated that TEER values and the expression of ZO-1, occludin, and Claudin-11 were markedly reduced in the presence of both the estradiol and fulvestrant-only group, indicating compromised barrier integrity (Figure 4I,J). Notably, taurine supplementation significantly upregulated TEER values and the expression of these proteins. These results revealed that taurine can rescue the antagonistic effects of fulvestrant on estradiol, suggesting that taurine could reverse the disruption in skin barrier function caused by estradiol deficiency (Figure 4I,J).

The above findings revealed the potential of taurine to act as a substitute for estradiol in restoring tight junction protein expression and collagen production impaired by estradiol deficiency.

### 3.5. Taurine Restored Skin Barrier Disruption in Sleep-Deprived (SD) Mice

**Figure 5:**
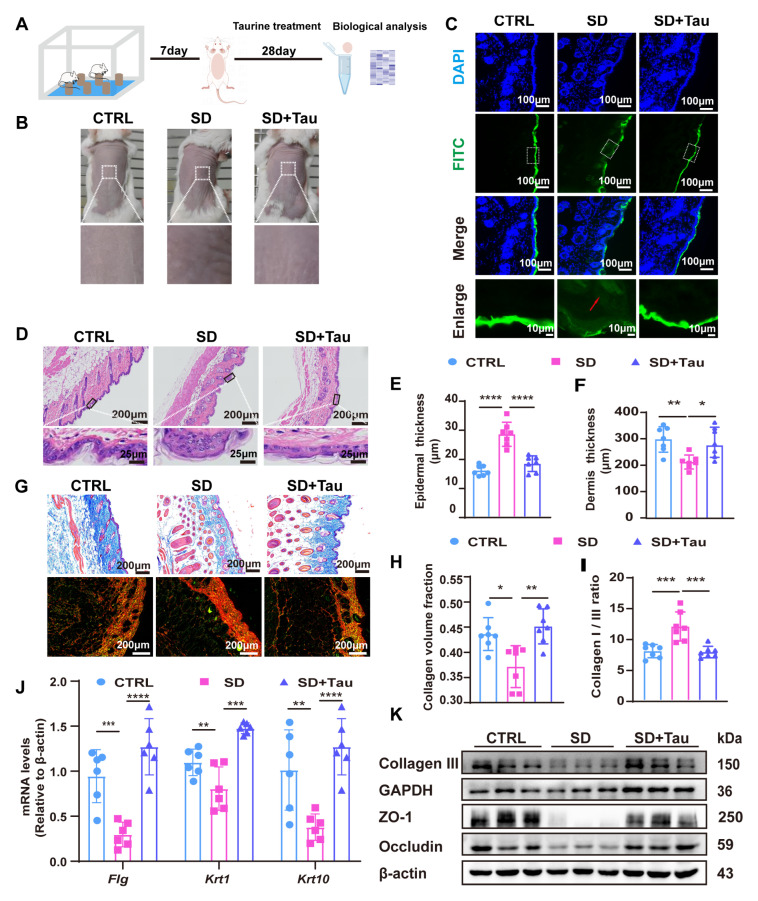
Taurine restored skin barrier disruption in sleep-deprived (SD) mice. (**A**). Schematic diagram of the animal experiment. (**B**). Changes in the skin phenotype of mice in different groups. (**C**). FITC (green) permeability test for skin barrier integrity. Nuclei were stained with DAPI (blue). The bottom panel showed a magnified view of the highlighted area. Local permeability was highlighted by the red arrow. Scale bars, 100 μm and 10 μm. (**D**). HE staining of skin tissue (n = 7). Scale bars, 200 μm and 25 μm. (**E**,**F**). Epidermal and Dermal Thickness Changes Measured by ImageJ software. The average of three fields of view was taken for each slice. (**G**). Masson staining and Sirius Red staining of skin tissue (n = 7). Scale bars, 200 μm. (**H**). Collagen volume fraction was counted using ImageJ. (**I**). The ratio of collagen I/collagen III was counted by ImageJ. (**J**). qRT-PCR to analyze mRNA level of *Flg*, *Krt1*, and *Krt10* (n = 6). (**K**). Western blot analysis and quantification of collagen III, ZO-1, occludin protein expression (n = 3). Data presented as means ± SD. * *p* < 0.05, ** *p* < 0.01, *** *p* < 0.001, **** *p* < 0.0001.

To further investigate whether taurine can serve as a substitute for estradiol in mitigating impairments in skin barrier function and dermal collagen synthesis induced by disruptions in the circadian rhythms of estradiol due to sleep deprivation, mice were subjected to sleep deprivation from 17:00 to 11:00 the next day, followed by a 6-h recovery period, over the course of seven days. Starting on the eighth day of sleep deprivation, a daily dorsal application of 100 μM taurine was topically administered for a total duration of 28 days (Figure 5A). At the conclusion of the 28-day treatment period, erythema and wrinkles were observed on the dorsal skin of sleep-deprived mice. In contrast, the application of taurine mitigated the appearance of erythema and wrinkle formation (Figure 5B). Then, FITC was utilized to assess skin barrier integrity. In the Ctrl group, where no treatment was applied, FITC was confined to the epidermal layer, indicating an intact skin barrier. Conversely, in the SD group, FITC penetrated into the dermis, suggesting a compromised skin barrier. However, treatment with taurine effectively prevented FITC from penetrating into the subcutaneous layer, indicating reconstruction of the skin barrier (Figure 5C).

The thickness of the epidermis layer increased from 15.27 μm in normal skin (control) to 26.42 μm in the SD groups. Notably, taurine significantly suppressed sleep deprivation-induced epidermal thickening. The epidermal thickness (17.36 μm) in the taurine treatment group resembled that of the normal control (15.27 μm) (Figure 5D,E). The thickness of the dermis layer decreased from 310.79 μm in normal skin to 232.31 μm in the SD groups. Taurine treatment significantly recovered the dermal thinning, further supporting its protective effect against sleep deprivation-induced skin damage (Figure 5D,F).

To assess the impact of taurine on collagen fibril integrity in mouse skin, Masson’s trichrome staining was utilized. Sleep deprivation led to reduced collagen deposition and disrupted fiber organization. However, taurine treatment enhanced collagen alignment and increased deposition (Figure 5G,H). Further analysis with Sirius red staining and quantification of type I (orange or red) and type III (green) collagen showed a significant elevation in their proportions in the SD group compared to the control. In contrast, taurine treatment effectively restored these collagen ratios (Figure 5G,I).

We then investigated the impact of taurine on the epidermal barrier by examining the expression of skin barrier-related proteins FLG, KRT1, and KRT10 in the dorsal skin. Sleep deprivation resulted in a decrease in the mRNA levels of *Flg*, *Krt1*, and *Krt10*, as well as a reduction in the protein expression of ZO-1, occludin, and collagen type III. Treatment with taurine restored the mRNA levels of *Flg*, *Krt1*, and *Krt10*, and the protein expression of ZO-1 and occludin, as well as collagen type III, indicating its potential role in mitigating the damage induced by sleep deprivation (Figure 5J,K).

### 3.6. Estradiol and Taurine Regulated the Expression of TJ Proteins and Collagen Production by Up-Regulating TMEM38B

**Figure 6:**
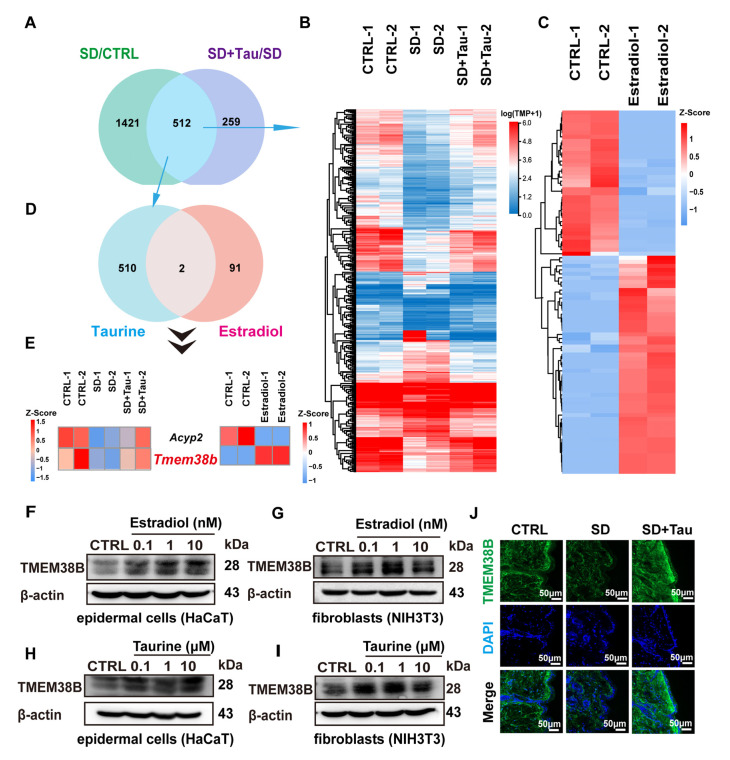
Estradiol and taurine regulated the skin barrier by up-regulating TMEM38B. (**A**). Venn diagram showing differential genes for SD/Control and SD + Tau/SD. Filtering conditions: Qvalue ≤ 0.05, |log2FC| ≥ 1. (**B**). Heat map analysis illustrating the expression patterns of 512 differential genes in a Venn diagram. The color scale indicated relative changes in protein expression, with red indicating up-regulation and blue indicating down-regulation. (**C**). Heat map analysis showed the expression patterns of 90 differential proteins after estradiol treatment. Filtering conditions: *p* < 0.05, FC > 1.2 was a significant up-regulation change threshold (the color patch is red), FC < 1/1.2 was a significant down-regulation change threshold (the color patch is blue). (**D**). Venn diagram assessing the intersection of 90 differential genes for estradiol treatment and 512 genes for taurine treatment. (**E**). Heat map analysis demonstrated the expression of two differentially expressed genes in Venn diagram in different groups. (**F**,**G**). Expression of TMEM38B was detected by Western blot after 24 h treatment with estradiol, respectively, in HaCaT and NIH 3T3 cells. (**H**,**I**). Expression of TMEM38B was detected by Western blot after 24 h treatment with taurine, respectively, in HaCaT and NIH 3T3 cells. (**J**). Immunofluorescence staining of TMEM38B in skin tissue (green). Nuclei were stained with DAPI (blue). Scale bars, 50 μm. The data were representative of at least three independent experiments and presented as means ± SD.

To investigate the molecular mechanisms by which taurine and estradiol enhance the skin barrier and collagen synthesis, we performed transcriptomic analysis on skin tissues from three groups: normal mice, sleep-deprived mice, and mice treated with taurine following sleep deprivation. The results revealed that 1933 genes were differentially expressed in the sleep deprivation group compared to normal mice, while 771 genes showed significant changes in the sleep deprivation group treated with taurine. Venn diagram analysis identified 512 overlapping genes between these two comparisons, and their expression patterns were visualized using a heat map (Figure 6A,B). KEGG enrichment analysis revealed that 25 genes were significantly associated with the calcium signaling pathway, ranking first among the enriched pathways, which suggested the pivotal role of taurine in calcium regulation (Figure S2).

Moreover, to identify the molecular targets of estradiol, we further treated cells with estradiol and performed quantitative mass spectrometry. This analysis identified 90 proteins with significant changes, of which 54 were upregulated and 36 were downregulated after estradiol treatment (Figure 6C). Venn diagram analysis of these 90 estradiol-responsive proteins with the 512 genes regulated by taurine treatment identified two overlapping genes: *Acyp2* and *Tmem38b* (Figure 6D,E). Notably, TMEM38B, also known as TRIC-B, is a transmembrane protein that functions as a cation-selective channel essential for maintaining rapid intracellular calcium (Ca^2+^) release. Western blot analysis demonstrated that estradiol and taurine upregulated TMEM38B protein levels in a dose-dependent manner, with the most pronounced effects observed at concentrations of 10 nM and 10 μM, respectively (Figure 6F–I). This result indicated that TMEM38B may be a common target for both taurine and estradiol (Figure 6F–I). Immunofluorescence staining of skin tissues also showed that TMEM38B protein levels were significantly reduced in the sleep-deprived mice compared to normal mice, but taurine treatment restored TMEM38B expression to levels comparable to those in normal mice (Figure 6J). These results suggested that TMEM38B may be involved in taurine- and estradiol-regulated tight junction-related proteins and collagen expression.

### 3.7. TMEM38B Affects Tight Junction-Related Proteins and Collagen Expression by Regulating Intracellular Calcium Levels

**Figure 7:**
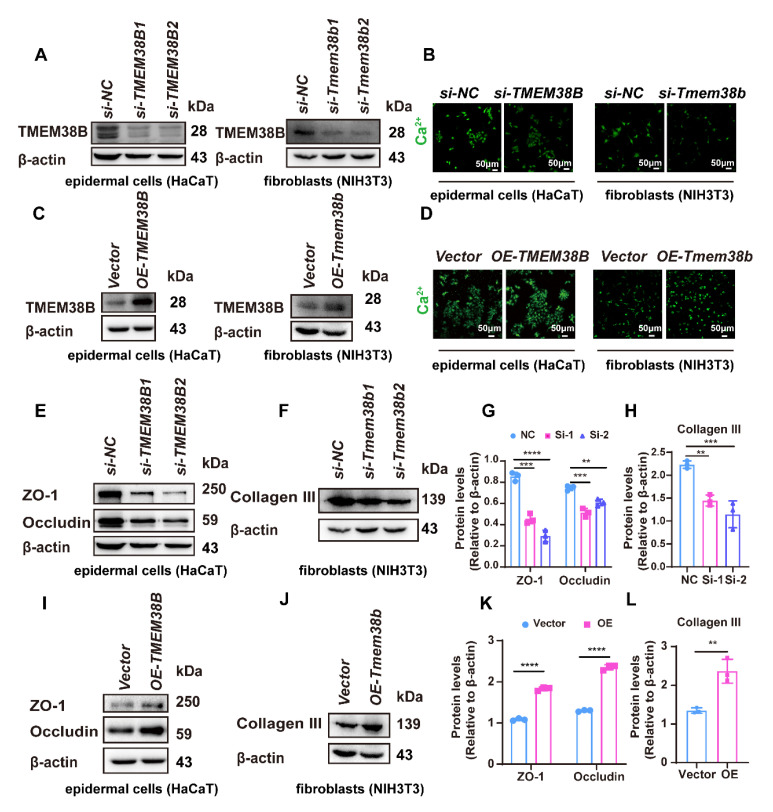
TMEM38B affects tight junction-related proteins and collagen expression by regulating intracellular calcium levels. (**A**). Knockdown of TMEM38B protein by siRNA was verified by Western blot. (**B**). Intracellular calcium concentration was detected by a calcium fluorescence probe (Calbryte^TM^520AM) in TMEM38b-deficient cells. (**C**). The overexpression of TMEM38B protein was verified by Western blot. (**D**). Calcium concentration was detected by Calbryte^TM^520AM in TMEM38b-overexpressed cells. (**E**,**F**). Protein expression of ZO-1, occludin, and collagen III was observed in TMEM38B-deficient cells. (**G**,**H**). The relative expression levels of proteins in (**E**,**F**) were quantified by ImageJ software. (**I**,**J**). Protein expression of ZO-1, pccludin, and collagen III was observed in TMEM38B-overexpressed cells. (**K**,**L**). The relative expression levels of proteins in (**I**,**J**) were quantified by ImageJ software. The data were representative of at least three independent experiments and presented as means ± SD. ** *p* < 0.01, *** *p* < 0.001, **** *p* < 0.0001.

Since TMEM38B plays a crucial role in regulating calcium homeostasis in cells, we examined whether TMEM38B controls calcium levels in epidermal cells and fibroblasts. We knocked down the expression of TMEM38B and stained intracellular calcium with Calbryte^TM^520AM dye to monitor changes in calcium levels. Compared to the control group, cells with reduced TMEM38B expression exhibited a significant decline in intracellular calcium concentration (Figure 7A,B). In contrast, overexpression of TMEM38B significantly increased the calcium levels in epidermal cells and fibroblasts (Figure 7C,D). These results suggested that TMEM38B is involved in regulating intracellular calcium levels in epidermal cells and fibroblasts.

We then examined whether intracellular calcium levels in epidermal cells and fibroblasts participate in regulating the expression of tight junction-related proteins (ZO-1, occludin, and Claudin-11) and collagen. We found that the downregulation of ZO-1, occludin and collagen III was observed in TMEM38B-silenced cells, while their expressions were enhanced in TMEM38B-overexpressed cells (Figure 7E–L). These results suggested that TMEM38B controls intracellular calcium levels and affects tight junction-related proteins and collagen expression.

### 3.8. Estradiol and Taurine Augment Tight Junction-Related Proteins and Collagen Protein Expression via Elevating Intracellular Calcium Levels by Targeting TMEM38B

**Figure 8:**
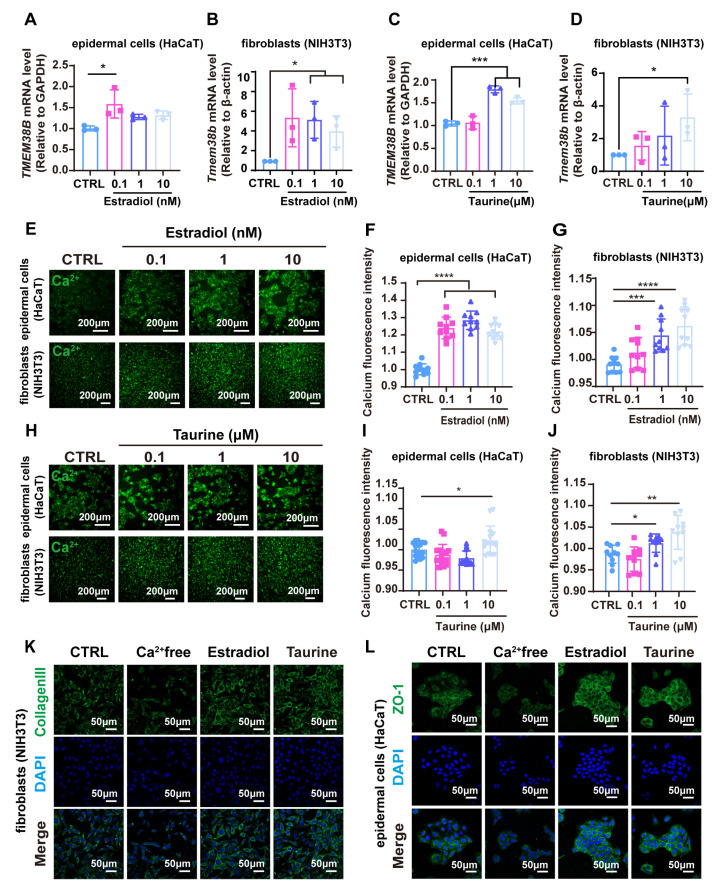
Estradiol and taurine augment tight junction-related proteins and collagen protein expression via elevating intracellular calcium levels by targeting TMEM38B. (**A**,**B**). Detection of mRNA levels of *Tmem38B* by qRT-PCR after estradiol treatment in HaCaT or NIH3T3. (**C**,**D**). Detection of mRNA levels of *Tmem38B* by qRT-PCR after taurine treatment in HaCaT or NIH3T3. (**E**). Calcium imaging using Calbryte^TM^520AM in HaCaT and NIH 3T3 cells treated with estradiol. (**F**,**G**). Fluorescence intensity of calcium ions in (**E**) was measured with a fully functional microplate reader. (**H**). Calcium imaging using Calbryte^TM^520AM in HaCaT and NIH 3T3 cells treated with taurine. (**I**,**J**). Fluorescence intensity of calcium ions in (**H**) was measured with a fully functional microplate reader. (**K**,**L**). Immunofluorescence of collagen III and ZO-1 under calcium ion-free medium, estradiol, and taurine treatment for 24 h. Nuclei were stained with DAPI (blue). Scale bars, 50 μm. The data were representative of at least three independent experiments and presented as means ± SD. * *p* < 0.05, ** *p* < 0.01, *** *p* < 0.001, **** *p* < 0.0001.

To further confirm whether TMEM38B is regulated by estradiol and taurine, we examined the levels of *Tmem38b* mRNA in epidermal cells and fibroblasts after treatment with either estradiol or taurine. Both estradiol and taurine significantly upregulated the expression of *Tmem38b* in these cell types (Figure 8A–D). Accompanying the increased expression of *Tmem38b*, we observed that both taurine and estradiol significantly enhanced Ca^2+^ levels in both epidermal cells and fibroblasts (Figure 8E–J).

To further elucidate the role of Ca^2+^ levels in regulating the expression of tight junction-related proteins ZO-1 and collagen, epidermal cells and fibroblasts were treated with calcium-free medium. Immunofluorescence analysis revealed that Ca^2+^ depletion significantly reduced the production of collagen III and ZO-1. In contrast, either taurine or estradiol treatments rescued the effect of calcium depletion, upregulating the expression of type III collagen and ZO-1 (Figure 8K,L).

Altogether, these results revealed taurine restored skin barrier function and mitigated collagen degradation by upregulating TMEM38B, increasing intracellular calcium ion levels, and promoting the expression of TJ-related proteins and type III collagen, thereby restoring skin homeostasis.

## 4. Discussion

Disruptions in circadian rhythm, such as sleep deprivation, have been shown to adversely affect skin barrier function and collagen production [12,14]. This study demonstrated that abnormal circadian rhythms in female mice led to alterations in estradiol rhythms, which subsequently impacted epidermal barrier integrity and collagen synthesis. Estradiol significantly enhanced the expression of TJ-related proteins and promoted collagen production. Additionally, the study revealed that taurine exerted a significant protective effect on skin issues resulting from circadian rhythm disturbances. Both estradiol and taurine elevated intracellular calcium ion levels by targeting TMEM38B, thereby promoting the expression of TJ proteins and collagen. The introduction of taurine represents a novel intervention strategy for addressing skin barrier function and collagen production disorders associated with circadian rhythm disruptions.

The secretion of estradiol is intricately mediated by the HPO axis and demonstrates notable circadian rhythmicity [17]. The gene expression of enzymes related to estradiol metabolism, such as CYP1A1 and CYP3A4, are regulated by biological clock genes, including *Per1* and *Per2*, and typically reach a peak or trough at specific time points, thereby influencing the metabolic rate of estradiol [36,37]. Additionally, estradiol receptors (ERα and ERβ) possess E-box elements that are subject to regulation by the circadian clock. The primary regulatory components of the circadian clock, *Clock* and *Bmal1*, can modulate the expression of various hormone receptors, including estradiol receptors [38]. An imbalance in the circadian clock disrupts the rhythmic expression of circadian clock genes, which notably impacts the timing and levels of estradiol secretion [15]. Research indicates that circadian clock disruption can result in metabolic rhythm disorders; for instance, the secretion rhythms of insulin and glucocorticoids are altered, which subsequently influences energy metabolism and fat storage, directly impacting estradiol synthesis [39,40]. Additionally, an imbalance in the circadian clock may cause changes in the expression of estrogen receptors. This alteration may stem from the dysregulation of circadian clock genes, which affects the transcriptional activity of the estradiol receptor gene [41]. Functional changes in estradiol receptors can lead to a diminished cellular response to estradiol, indirectly resulting in reduced availability of it. The deficiency of estradiol negatively impacted the homeostasis of dermal cells, resulting in considerable declines in skin health, characterized by fibroblast dysfunction, decreased collagen and elastin content, and barrier permeability issues, which ultimately led to skin thinning, dryness, wrinkles, and atrophy [42,43,44]. Studies have shown that ovariectomy in young mice resulted in diminished skin tensile strength, reduced fibrillin-1 expression, and degradation of elastic fibers; however, administration of 17β-estradiol reversed the decline in skin mechanical properties and stimulated elastin synthesis driven by fibroblasts [45]. In a 28-day chronic sleep restriction (SR) mouse model, reduced sleep was shown to degrade tight junction proteins and induce oxidative stress in the skin [46]. Our experiments demonstrated that estradiol promoted the expression of tight junction-related proteins in epidermal cells and enhanced collagen synthesis in fibroblasts. In a mouse model of circadian rhythm disruption induced by sleep deprivation, estradiol levels exhibited a loss of circadian pattern and a significant decrease, contributing to the observed skin wrinkling, altered skin thickness, and diminished collagen deposition in the model group. Given the significance of estrogen in maintaining skin homeostasis, estrogen replacement therapy (ERT) has been demonstrated to enhance levels of skin elastin and collagen, thereby serving as a protective measure for postmenopausal skin [47,48,49]. However, its clinical application remains a subject of debate, as data from a large cohort study associated ERT with an elevated risk of breast cancer [25]. Consequently, the search for safer alternatives has intensified, leading to a growing interest in phytoestrogens and tissue-selective pharmaceuticals, such as selective estrogen receptor modulators (SERMs) [50].

As a naturally occurring amino acid in the body, taurine is expected to have minimal side effects, with toxicity studies showing no genotoxic or carcinogenic effects [51]. A human trial has shown that daily consumption of up to 1000 mg of taurine is well tolerated without adverse effects [52]. Taurine has been demonstrated to enhance filaggrin expression in three-dimensional cultured epidermis, thereby contributing to the production of natural moisturizing factor (NMF), which effectively moisturizes the skin [53]. Additionally, taurine has been shown to stimulate the synthesis of lipids, including cholesterol, ceramides, and fatty acids, thereby improving skin barrier function [54]. Our experiments indicated that taurine increased the trans-epithelial electrical resistance (TEER) value of epidermal cells and elevated the expression of tight junction proteins such as ZO-1, occludin, and Claudin 11, while also promoting the synthesis of type III collagen in fibroblasts. Furthermore, taurine exhibited a significant therapeutic effect on sleep-deprived mice, effectively restoring the skin barrier compromised by decreased levels of *Flg*, *Krt1*, *Krt10*, and tight junction-associated proteins, while promoting the orderly arrangement and increased deposition of collagen. These findings offer a valuable reference for treatment strategies addressing skin issues related to sleep deprivation and disruptions in circadian rhythms. Well-established skin barrier protectors, such as hyaluronic acid, ceramides, and niacinamide, are widely recognized for their anti-wrinkle, anti-aging, and moisturizing properties [55,56,57]. Future research should investigate whether the combination of taurine with these ingredients can provide additive or synergistic benefits in enhancing skin barrier function. Additionally, based on the biochemical properties of taurine, optimizing its delivery system to improve transdermal absorption kinetics and local bioavailability could be a direction worth further investigating.

We further identified TMEM38B as a key protein jointly targeted by estradiol and taurine through cross-omics analysis. Both taurine and estradiol promoted the expression of TMEM38B in epidermal cells and fibroblasts. Furthermore, taurine was able to reverse the reduction of TMEM38B protein expression caused by estrogen deficiency in sleep-deprived mice. TMEM38B plays a crucial role in regulating calcium ion release and homeostasis within the endoplasmic reticulum (ER) [58]. Our experiments demonstrated that reduced TMEM38B expression significantly decreased intracellular calcium abundance. Conversely, the overexpression of TMEM38B led to a marked increase in intracellular calcium levels, further confirming the essential role of TMEM38B in the regulation of calcium ions in both epidermal and fibroblast cells. Research indicated that a deficiency in TMEM38B resulted in impaired Ca^2+^ release from the ER, decreased calcium flux, and alterations in the expression and activity of calcium-binding partner proteins, as well as various collagen-modifying enzymes associated with the ER, thus impacting collagen synthesis and secretion [59]. This aligned with our findings that knockdown of *Tmem38b* led to decreased collagen III expression in fibroblasts, while increased TMEM38B expression correlated with enhanced collagen III synthesis. Notably, we observed that the deletion of TMEM38B resulted in significant down-regulation of tight junction proteins (ZO-1 and occludin), whereas overexpression of TMEM38B promoted the expression of these proteins. These results underscore the critical role of TMEM38B as a co-targeting protein of estradiol and taurine in barrier protection and collagen synthesis.

The epidermal calcium gradient is crucial for epidermal differentiation and skin barrier formation, with intracellular Ca^2+^ concentrations increasing in a gradient that peaks in the granular layer. Most intracellular Ca^2+^ is stored in the endoplasmic reticulum (ER) and regulated by calcium channels or pumps [60]. In response to extracellular stimuli, Ca^2+^ is released from the ER lumen into the cytoplasm through inositol trisphosphate (IP3) and ryanodine receptors. Subsequently, cytoplasmic Ca^2+^ is transported back to the ER via the sarco/endoplasmic reticulum Ca^2+^ ATPase (SERCA) pump [59]. Research has indicated that mutations in the SERCA2 protein can disrupt Ca^2+^ concentrations in cells, ultimately leading to abnormal epidermal differentiation [61]. Our study found that the loss of TMEM38B may significantly contribute to impaired skin barrier function. Throughout the differentiation of keratinocytes, calcium influences the expression of almost all genes responsible for coding differentiation-specific proteins. Various precursor proteins such as loricrin (LOR), involucrin (INV), filaggrin (FLG), and keratin (KRT) undergo covalent cross-linking into the cornified envelope by transglutaminase 1, and this process is dependent on calcium levels. Elevated calcium concentration activates intracellular protein kinase C (PKC), thereby inducing differentiation markers in keratinocytes [62]. Consistent with our experimental results, the differentiation markers *Flg*, *Krt1*, and *Krt10* detected in the skin of sleep-deprived mice were significantly lower than those in the control group. However, taurine treatment was able to upregulate these markers by enhancing the expression of TMEM38B and promoting Ca^2+^ concentration. This restoration of marker expression facilitated the normal process of skin barrier formation. Additionally, the recovery of tight junction proteins ZO-1 and occludin was also observed, further illustrating the crucial role of calcium ions in barrier protection. It is noteworthy that elevated intracellular Ca^2+^ levels also triggered signaling pathways related to fibroblast proliferation and served as key regulators of collagen synthesis [63]. Our experiments revealed that taurine could restore the expression of TMEM38B in sleep-deprived mice, thereby repairing the skin barrier damaged by Ca^2+^ deficiency. However, the underlying molecular mechanisms of how TMEM38B regulates calcium ion levels and how Ca^2+^ influences tight junction proteins and collagen expression warrant further investigation. Additionally, it would be advantageous to examine whether taurine provides protective effects in other dermatological conditions, such as eczema and acne.

These findings suggest that taurine may serve as a viable alternative to estradiol, effectively improving skin homeostasis disrupted by sleep deprivation while mitigating the potential risks associated with exogenous estrogen supplementation. Moreover, these results not only provide preliminary insights into the protective mechanisms of taurine against skin impairments induced by sleep deprivation but also indicate its potential application in treating skin conditions related to estrogen imbalances, such as skin aging in menopausal women. However, unanswered questions present specific limitations in this study. Although the study identifies the important regulatory role of TMEM38B in taurine-mediated effects, the upstream molecular mechanisms still need to be further explored. In addition, all experiments were conducted using in vitro cell models and mouse models. While these findings are promising, they have yet to be validated in the context of more complex human skin. To support the clinical application of taurine, future studies should explore its dose–response relationship across a range of concentrations, using human skin models or through long-term clinical trials to evaluate both efficacy and safety.

## 5. Conclusions

**Figure 9:**
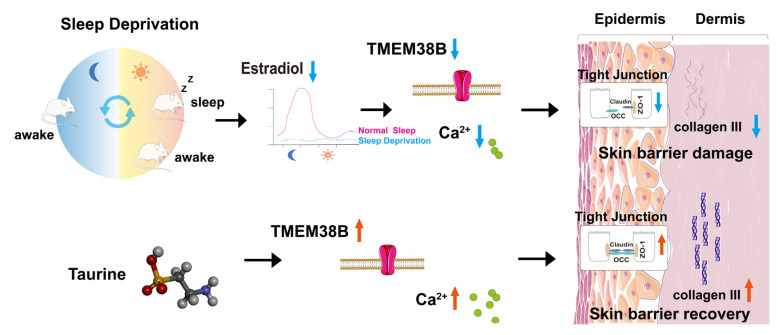
Schematic diagram illustrating the mechanism by which taurine alleviates sleep deprivation-induced skin barrier damage. Sleep deprivation disrupts the circadian rhythm of estrogen and impairs skin barrier integrity. Taurine restores skin homeostasis by upregulating TMEM38B, which modulates intracellular calcium ion concentrations.

This study reveals that chronic sleep deprivation disrupts estradiol circadian rhythms, resulting in compromised epidermal barrier function and diminished dermal collagen synthesis. Taurine effectively mitigates these impairments by upregulating TMEM38B, a trimeric cation channel essential for maintaining intracellular calcium homeostasis. The resulting elevation in calcium levels promotes the expression of tight junction proteins (ZO-1 and occludin) in keratinocytes and stimulates type III collagen production in fibroblasts, thereby restoring skin homeostasis (Figure 9). These findings suggest that taurine might serve as a promising alternative to exogenous estrogen for mitigating sleep deprivation-induced skin damage and provide preliminary insights into the protective mechanisms. Importantly, taurine not only effectively improves skin homeostasis disrupted by sleep deprivation but also reduces the potential risks associated with exogenous estrogen supplementation. This suggests that taurine could be considered a potential therapeutic option for addressing skin aging and dysfunction associated with circadian rhythm disturbances.

## Figures and Tables

**Table 1:** Primers used in qRT-PCR.

Name	Sense Primers (5′–3′)	Anti-Sense Primers
*Per1*	CAACGTGATGGGTCGCTTTC	GTAAAGGGGTCGTGTTCGCT
*Per2*	GCGCACCAAGTGACGGG	ACTTGGGGAGAAGTCCACGTA
*Bmal1*	GGCCCAAAGAGGACTCATCC	GCGATGACCCTCTTATCCTGT
*Flg*	TCCTGGAAAGCATCACTAGCA	TGTCTTGGTCATCTGGATTCTTCA
*Krt1*	GTTGCAAGAAGCAGATCTCCCA	AGTGCTTTCTCACCACGCTG
*Krt10*	AGTGGTACGAGAAGCATGGC	GCACGTTGGCATTGTCAGTT
*TMEM38B *	GTGCCCTCTCCTACTCCTCA	GCTGCTCCCGGCTGA
*GAPDH *	CACCATCTTCCAGGAGCGAG	AGAGGGGGCAGAGATGATGA
*Tmem38b*	GCCCTCGTGACCTAGTTTCC	CCGCCTACTATTTTCCACGTC
*β-actin *	GAGCGCAAGTACTCTGTGTG	AACGCAGCTCAGTAACAGTC

**Table 2:** Antibody information.

Antibody	Source	Item Number	Working Dilution
ZO-1	Abcam	Ab221547	1:100 (IF)–1:1000 (WB)
Occludin	Abcam	Ab216327	1:1000 (WB)
Claudin-11	Invitrogen	364500	1:100 (IF)–1:1000 (WB)
Collagen III	Abcam	EPR17673	1:1000 (WB)
Collagen I	Abcam	Ab21286	1:1000 (WB)
Collagen I	Abcam	Ab260043	1:1000 (WB)
TMEM38B	Proteintech	19919-1-AP	1:100 (IF)–1:500 (WB)
GAPDH	Abcam	ab245355	1:5000 (WB)
β-actin	Abcam	ab8226	1:5000 (WB)

**Table 3:** Primers used in siRNA.

Name	Sense Primers (5′–3′)	Anti-Sense Primers (5′–3′)
si-*TMEM38B*-1	CCAUUGAAGUUUCUUGCAATT	UUGCAAGAAACUUCAAUGGTT
si-*TMEM38B*-2	GGAUAGUCAUGAUAGCUAUUGTT	CAAUAGCUAUCAUGACUAUCCTT
si-*Tmem38b*-1	CCAGGGUUAUUCAUAUCAATT	UUGAUAUGAAUAACCCUGGTT
si-*Tmem38b*-2	CCUGGAUAGUCAUGAUAGUTT	ACUAUCAUGACUAUCCAGGTT

## Data Availability

The data presented in this study are available on request from the corresponding authors.

## Supplementary Materials

The following supporting information can be downloaded at https://www.mdpi.com/article/10.3390/cells14100727/s1, Figure S1: CCK8 detection of taurine cytotoxicity; Figure S2: KEGG pathway enrichment analysis of taurine treatment.
