# Supplementary material for: Taurine Prevents Impairments in Skin Barrier Function and Dermal Collagen Synthesis Triggered by Sleep Deprivation-Induced Estrogen Circadian Rhythm Disruption
Source: Cells. 2025 May 16;14(10):727. doi: 10.3390/cells14100727 (PMC12110213; doi:10.3390/cells14100727)
Supplement: Supplementary file 1 [file cells-14-00727-s001.zip › cells-3595309-supplementary.pdf]

Supplementary Figures:

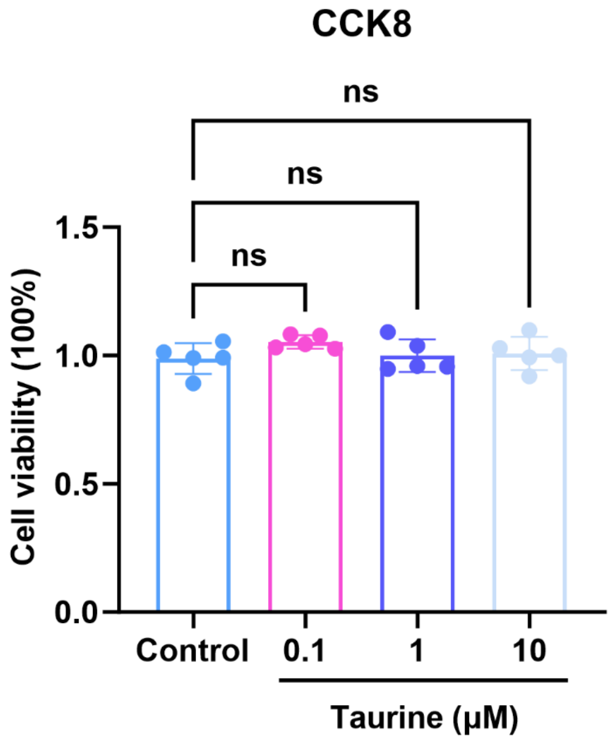

Figure S1. CCK8 detection of taurine cytotoxicity. HaCaT cells were seeded in 96-well plates and treated with taurine at concentrations of 0.1, 1, and 10 μM for 24 hours, with 8 replicate wells per concentration. Cell viability was assessed using the CCK-8 assay kit (HY-P0093) by measuring absorbance at 450 nm (n=5). The data were presented as means± SD. ns = not significant.

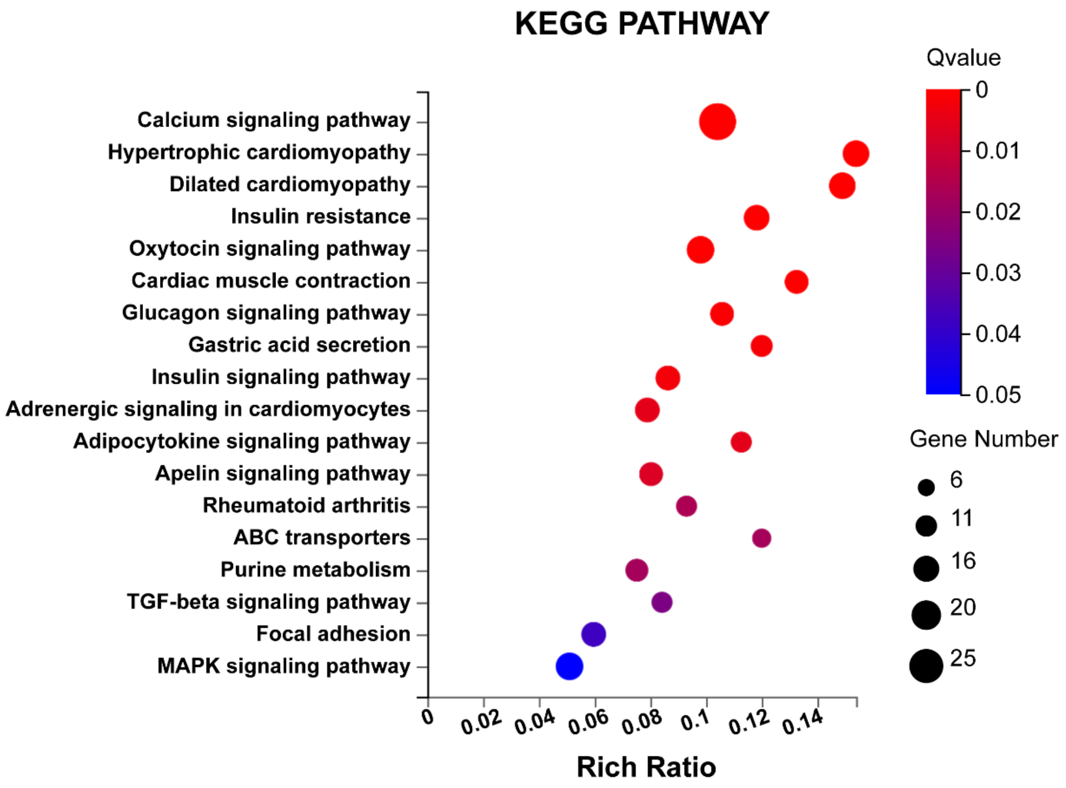

Figure S2. KEGG pathway enrichment analysis of taurine treatment. The analysis identified significantly enriched biological pathways associated with 512 differentially expressed genes, derived from the intersection of the SD/Control and SD+Tau/SD comparison groups. Bubble size indicates the number of genes involved in each pathway, while color represents statistical significance (a smaller Q-value indicates greater enrichment significance). Pathways with a Q-value ≤ 0.05 were considered significantly enriched. The top 18 enriched pathways are presented.
